# Supplementary figures and images for: Comprehensive analysis of DNA methylation patterns in recurrent miscarriage: imprinted/non-imprinted genes and their regulation across sperm and fetal-maternal tissues
Source: PeerJ. 2025 Oct 7;13:e20125. doi: 10.7717/peerj.20125 (PMC12513379; doi:10.7717/peerj.20125)

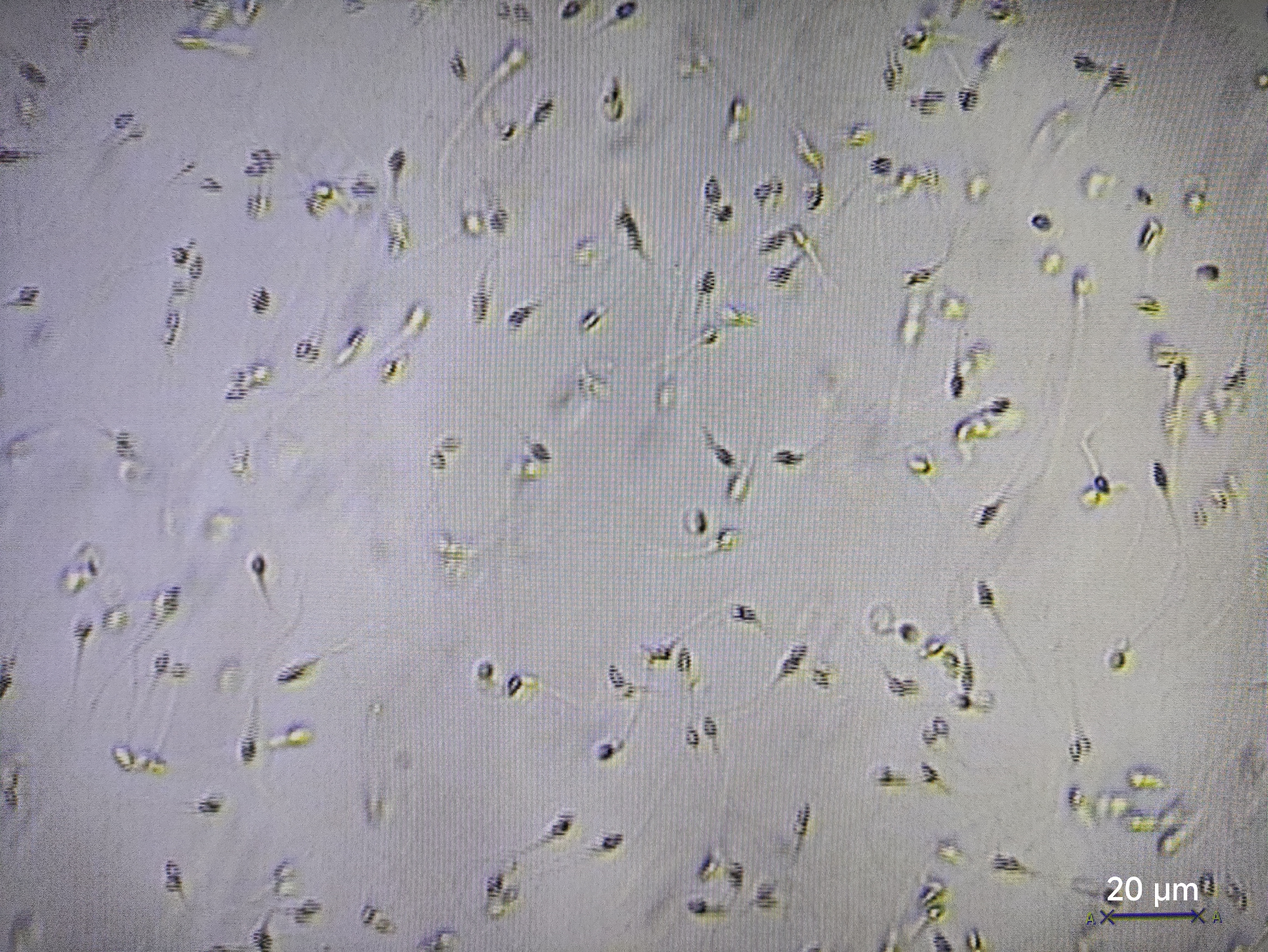

Supplement: Supplemental Information 1 — Motile sperm isolated from semen samples show characteristic morphology with intact flagella (Scale bar: 20 μ m). [file peerj-13-20125-s001.jpg]

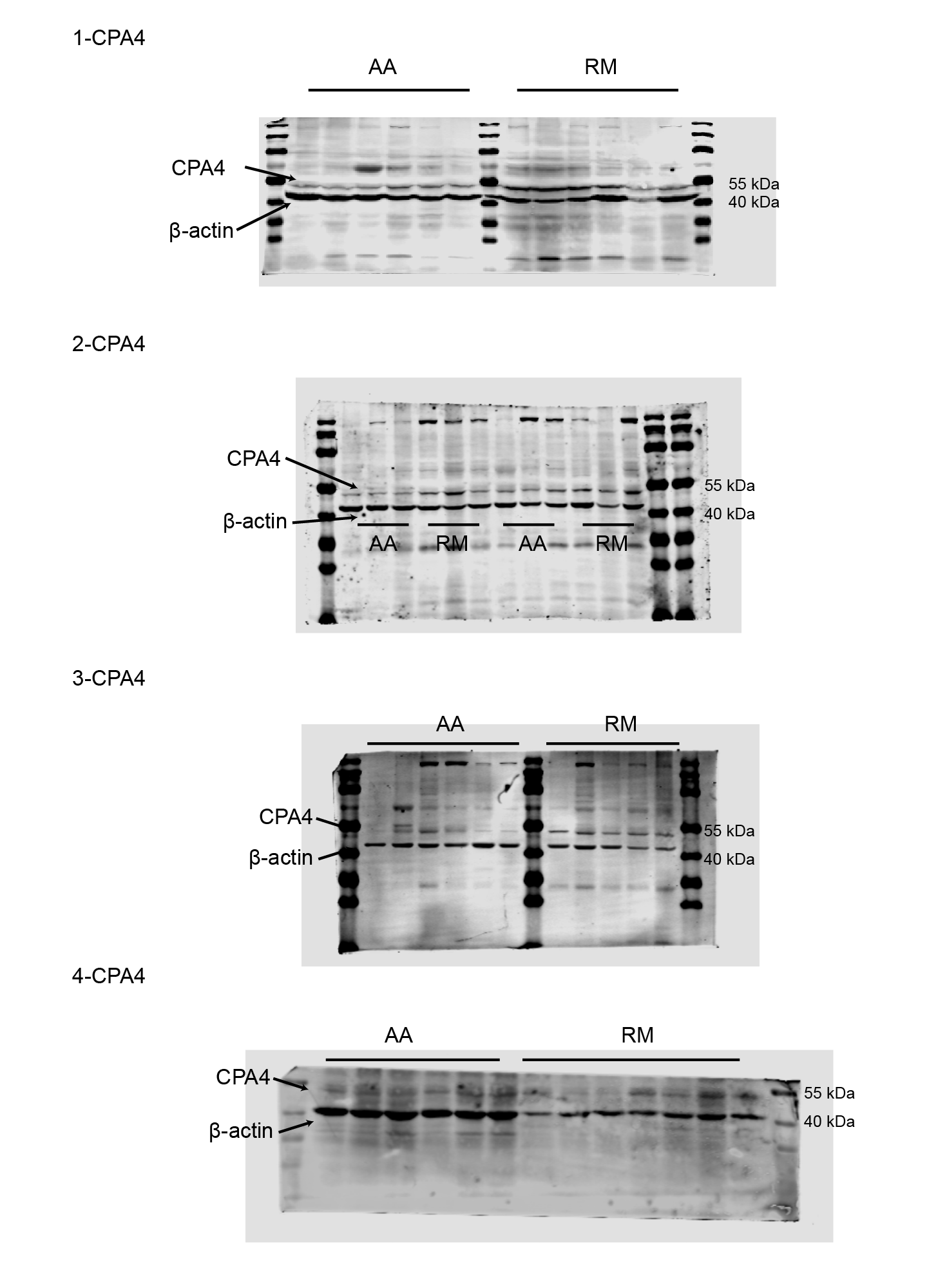

Supplement: Supplemental Information 8 [file peerj-13-20125-s008.zip › CPA4-01.tif]

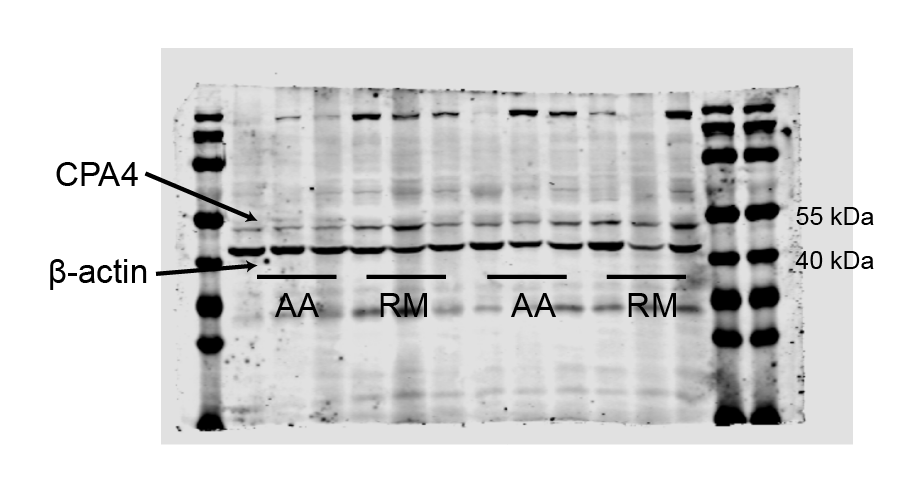

Supplement: Supplemental Information 8 [file peerj-13-20125-s008.zip › Figure5-ACTIN-CPA4-01.tif]

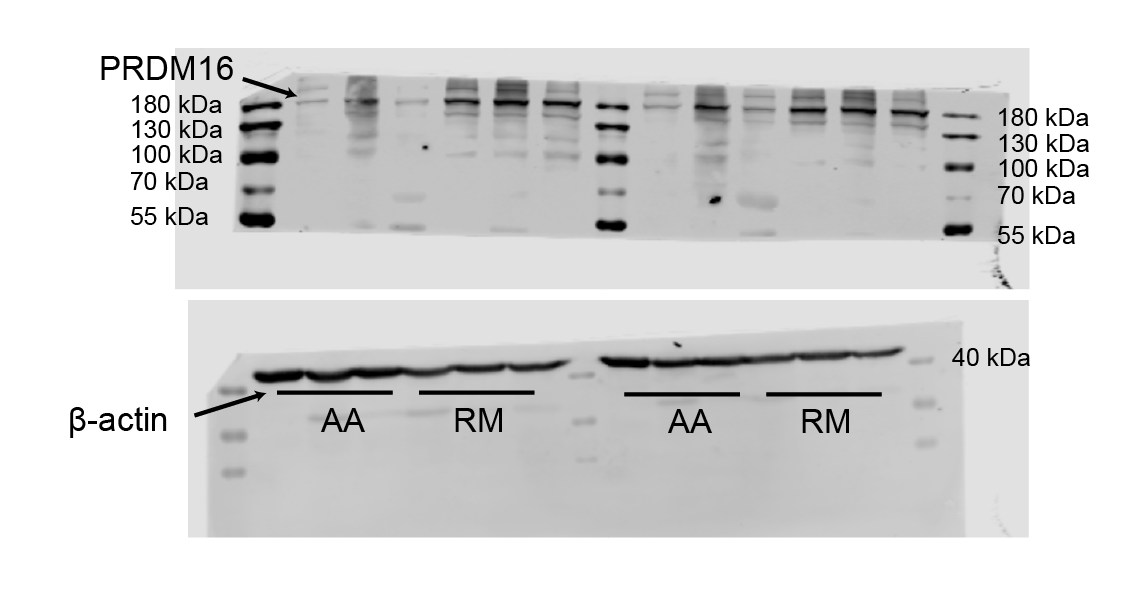

Supplement: Supplemental Information 8 [file peerj-13-20125-s008.zip › Figure5-PRDM6-ACTIN-01.tif]

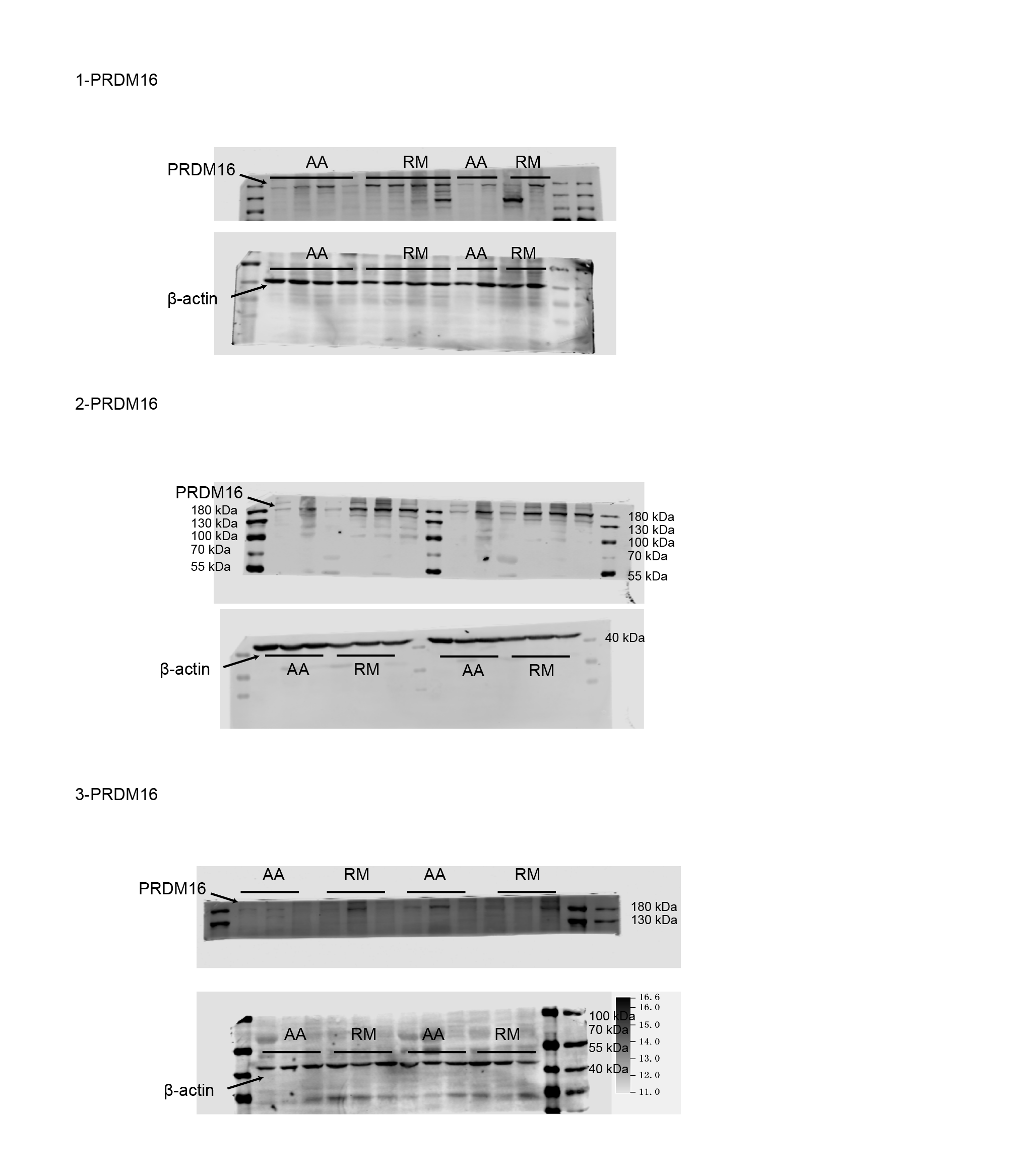

Supplement: Supplemental Information 8 [file peerj-13-20125-s008.zip › PRDM16-01.tif]
